# Editorial: Innovative treatments for neuro-psychiatric diseases

**DOI:** 10.3389/fnins.2023.1247681

**Published:** 2023-07-07

**Authors:** Lucia Gozzo, Edoardo Spina, Filippo Drago

**Affiliations:** ^1^Clinical Pharmacology Unit, Regional Pharmacovigilance Centre, Azienda Ospedaliero Universitaria Policlinico “G. Rodolico–S. Marco”, Catania, Italy; ^2^Department of Clinical and Experimental Medicine, University of Messina, Messina, Italy; ^3^Department of Biomedical and Biotechnological Sciences, University of Catania, Catania, Italy

**Keywords:** neuropsychopharmacology, pharmacological targets, innovative treatments, rare diseases, unmet needs

Neuropharmacology represents one of the therapeutic areas with the greatest number of molecules in development, reflecting research advances in the understanding of the basis of the diseases useful for promoting potentially innovative interventions (Pankevich et al., 2014; Gozzo et al., 2021a). However, neuropsychiatric conditions historically have been among the most difficult for which developing effective and safe new therapies, due to the complexity of pathophysiology and clinical presentation; therefore, curative treatments for important diseases, such as neurodegenerative diseases, are still lacking (O'Donnell et al., 2019; EC, 2020). Recognizing a true innovation can accelerate the development and adoption of valuable treatment options for neuropsychiatric disorders, encouraging their prioritization to make them quickly available for patients with high unmet clinical needs.

This Research Topic collects updated evidence about innovation for neuropsychiatric diseases, characterized by a major impact on the life expectancy and the quality of life of patients but also on the healthcare system and the society.

Major Depressive Disorder (MDD) still represents a major cause of morbidity, disability, and mortality worldwide (Trivedi, 2016) and several patients do not remit from the disease, even after multiple pharmacological and non-pharmacological treatment lines (Caldiroli et al., 2021). Therefore, the identification of innovative and effective treatment strategies represents a current challenge for research in neuropharmacology.

Dong et al. explored the effect of neferine (Nef) in a mouse model of chronic stress-induced depression. Mice were randomly assigned to the control group, the Nef group, and the fluoxetine (Flu) group. The study showed a significant decrease in the immobility time in the despair test, a significant increase in the levels of dopamine (DA), serotonin (5-HT), and norepinephrine (NE), and reduced brain damage of depressed mice with both Nef and Flu administration. The anti-depressive like effect of Nef can be mediated by the regulation of 5-HT/NE/DA reuptake but also by the modulation of the intestinal flora composition, in particular the abundance of Lactobacillus, underlying the critical role of the brain-gut-microbial axis.

Antipsychotics currently used to treat schizophrenia can help to manage the positive symptoms of the disorder but have minimal impact on the cognitive and negative symptoms (Maroney, 2022). Thus, these patients need innovative products to improve disease management.

Wang et al. studied potent trace amine-associated receptor 1 (TAAR1) agonists, which inhibit dopamine D2-like receptors and activates 5-HT1A receptors by regulating monoamine transmission and could be used as potential new compounds for the treatment of schizophrenia. They used molecular docking and molecular dynamics (MD) simulations to identify TAAR1 agonists. Then they assessed the potential antipsychotic effects of two selected agonists in a behavioral model of schizophrenia, demonstrating their potential beneficial effect.

Liu et al. focused on the role of long non-coding RNAs (LncRNAs) in the pathogenesis of epilepsy, a major public health issue which needs more effective and safe therapies. LncRNAs are no coding RNAs longer than 200 nucleotides, involved in a lot of pathophysiological processes. The role of these molecules in epileptogenesis can be linked to neuroinflammation, apoptosis, and transmitter balance (Kumar et al., 2022). The available antiseizure medications do not alter the long-term prognosis of the disease, and their long-term use is associated with many adverse effects. LncRNAs represent potential targets to develop innovative treatments for these patients.

Finally, Gozzo et al. analyzed the assessment of therapeutic value issued by European countries for innovative drugs approved for multiple sclerosis (MS). In Europe, all new drugs to treat neurodegenerative diseases as well as autoimmune and other immune dysfunctions must be authorized by the European Medicines Agency (EMA) via the Centralized Procedure (EMA, 2020; Gozzo et al., 2021a,b). Then, each Country is responsible for pricing and reimbursement decisions made according to the assessments performed by their own health technology assessment (HTA) bodies (van Nooten et al., 2012).

The therapeutic armamentarium for the treatment of multiple sclerosis (MS) has highly expanded in recent years, in particular for relapsing—remitting disease (RRMS) (Brancati et al., 2021; Brummer et al., 2022).

Disease-modifying therapies (DMTs) available for the treatment of MS demonstrated to reduce the inflammatory activity and relapse rate, but the efficacy on disability progression is limited.

The study showed no agreement among European HTA bodies on the therapeutic value of medicines authorized for MS in the reference period. Moreover, most drugs were associated to an “additional benefit not proven/no clinical improvement”, demonstrating the need for new effective and safe therapeutic options, especially for some forms and clinical settings of the disease.

## Author contributions

LG wrote the manuscript. ES and FD revised and approved the final draft. All authors contributed to the article and approved the submitted version.
